# In Vivo Study on the Effects of Xiaoaiping on the Stemness of Hepatocellular Carcinoma Cells

**DOI:** 10.1155/2019/4738243

**Published:** 2019-06-23

**Authors:** Jing Zhan, Liang-liang Shi, Yan Wang, Bai Wei, Sheng-li Yang

**Affiliations:** ^1^Division of Oncology, Liyuan Hospital, Tongji Medical College, Huazhong University of Science and Technology, Wuhan, 430077, China; ^2^Cancer Center, Union Hospital, Tongji Medical College, Huazhong University of Science and Technology, Wuhan, 430022, China

## Abstract

**Aims:**

The aim of this study was to examine the effects of Xiaoaiping on the stemness of hepatocellular carcinoma (HCC) cells* in vivo* and to investigate the underlying molecular mechanism.

**Methods:**

A subcutaneous xenograft nude mouse model was established using Hep3B-derived HCC cells. The mice were randomly assigned to the 100 mg/kg Xiaoaiping or 100 *μ*L/20 g normal saline (control) groups (n =3/sex/group) for daily intragastric administration for 14 days. The tumor size was closely monitored during the dosing phase. After the treatment period, the tumor tissues were weighed and harvested for mRNA and protein isolation. qPCR and Western blotting were used to evaluate the expression of cancer stemness markers (epithelial cell adhesion molecule [EpCAM], cluster of differentiation [CD13], CD90, aldehyde dehydrogenase 1 [ALDH1], CD44, and CD45), totipotency factors (sex determining region Y-box 2 [Sox2], Nanog, and octamer-binding transcription factor 4 [Oct4]), and genes involved in the Notch, Wnt/*β*-catenin, Hedgehog, and Hippo signaling pathways.

**Key Findings:**

The tumor size and weight were significantly reduced in the nude mice treated with 100 mg/kg Xiaoaiping when compared with the controls. The Xiaoaiping effects on the stemness markers and totipotency factors included decreased expression of EpCAM, CD24, CD47, Sox2, Oct4, and sal-like protein 4 (SALL4), as well as increased expression of CD13 and ALDH1. In addition, Xiaoaiping inhibited the Hippo, Wnt, and Hedgehog signaling pathways.

**Conclusion:**

Xiaoaiping significantly inhibited the growth of HCC xenograft in nude mice. These antitumor effects may be mediated by modulating the expression of multiple stemness markers and totipotency factors and inhibition of the Hippo, Wnt, and Hedgehog signaling pathways.

## 1. Introduction

Liver cancer is the fifth most common malignancy in the world and the third leading cause of cancer mortality in humans [1]. In China, liver cancer is the second and third leading cause of cancer deaths in men and women, respectively [2]. Less than 20% of the liver cancer patients are diagnosed at early stages and could be radically cured by surgery, liver transplantation, or radiofrequency ablation. Most patients with liver cancer are diagnosed at an advanced stage, and the median survival time of liver cancer patients is only 3-6 months [3]. Therefore, development of effective antitumor therapies for liver cancer remains to be an unmet medical need. In recent years, more and more traditional Chinese medicines (TCM) are being used in antitumor therapies, especially in adjuvant therapies for advanced malignant tumors. Certain TCM have been used in combination with classic chemotherapy regimens to alleviate adverse effects. Some TCM are able to reverse chemoresistance and enhance the therapeutic effects of chemotherapies [4].

Cancer stem cells (CSCs), also known as tumor-initiating cells, are a class of cells capable of self-renewal and differentiation into various nonstem cells. They play important roles in tumor growth, metastasis, recurrence, and drug resistance [5]. Tumor stemness has been identified in a variety of cancers, including liver cancer, lung cancer, ovarian cancer, colon cancer, prostate cancer, pancreatic cancer, and melanoma [5]. Liver cancer stem cells (LCSCs) have been discovered in liver cancer tissues [6]. Similar to normal adult stem cells, CSCs maintain their self-reproducing ability during ontogenetic development and tissue homeostasis through activating multiple stem cell signaling pathways and inhibiting the differentiation-related signaling pathways [7]. The stemness of liver cancer is characterized by several stem markers and totipotency factors and regulated via a few signaling pathways. The major stem markers in liver cancer include CD133, EpCAM, CD13, CD90, ALDH1, CD44, and CD45. The totipotency factors related to liver cancer include Sox2, Nanog, Oct4, and SALL4. The stemness signaling pathways consist of the Notch, Wnt/*β*-catenin, Hedgehog, and Hippo signaling pathways [7].

Xiaoaiping is the extract from the root of the TCM Marsdenia tenacissima, which has been reported to have anticancer effect. It has been reported that the combination of Xiaoaiping and cisplatin-based chemotherapy significantly enhances the anticancer efficacy in patients [8]. Moreover, the combination of Xiaoaiping and classic chemotherapies exerts an enhanced therapeutic effect against malignant tumors [9]. Studies have shown that Xiaoaiping is safe and effective for patients with advanced cancer when administered in combination with targeted drugs and chemotherapeutic drugs, and it does not increase the toxicity or side effects of chemotherapy [10].

Xiaoaiping has also been reported for potent antitumor effects against liver cancer in patients [11]. Han et al. showed that Xiaoaiping inhibited the in vitro metabolism of gefitinib by interfering with human liver enzymes CYP3A4 and CYP2D6, and, therefore, the combination therapy of Marsdenia tenacissima and gefitinib produced an improved antitumor effect [12]. However, there are few mechanistic studies on the anti-HCC effects of Xiaoaiping. Therefore, our present study established a subcutaneous xenograft nude mouse model using human HCC cells to investigate the Xiaoaiping effects on the tumor growth and expression patterns of LCSC surface markers, totipotency factors, and genes related to stemness signaling pathways.

## 2. Materials and Methods

### 2.1. Materials

The human HCC cell line, Hep3B cells, was purchased from the China Center for Type Culture Collection (CCTCC). Twelve BALB/C nude mice (4-5 weeks of age and weighing 20±2 g; 6 males and 6 females) were housed under specific-pathogen-free (SPF) conditions. Xiaoaiping (60 *μ*g/mL) was purchased from Nanjing Sanhome Pharmaceutical Co. Ltd. (China). Roswell Park Memorial Institute (RPMI) 1640 medium, fetal bovine serum (FBS), and trypsin were obtained from HyClone Laboratories Inc. (USA). Normal saline was ordered from MP Biomedicals (USA). TRIzol, SYBR® Green PCR Master Mix, and PCR primers were purchased from Life Technologies (USA). The major instruments used in this study included a carbon dioxide (CO2) incubator (Harris Corporation, USA), quantitative fluorescence PCR system (Ambion Inc., USA), electrophoresis apparatus (Bio-Rad Laboratories, USA), and high-speed refrigerated centrifuge (Heraeus Instruments, Germany).

### 2.2. Establishment of a Subcutaneous Xenograft Nude Mouse Model with Human HCC Cells

After resuscitation, the Hep3B cells were cultured in RPMI 1640 containing 10% FBS at 37°C in an atmosphere of 5% CO2. The culture medium was changed every 2 to 3 days, and the cells were passaged every 5 to 6 days. After three passages, the logarithmically growing cells were trypsinized, harvested, counted, and diluted in phosphate buffered saline (PBS) to a density of 1×10^7^ cells/mL. Using an insulin syringe, 0.2 mL of the cell suspension was slowly injected into the subcutaneous tissues on the back of the nude mice near the right axilla. After injection, the mice were returned to the cages and housed under sterile conditions. All the animal experiments were reviewed and approved by the Institutional Animal Care and Use Committee of Huazhong University of Science and Technology, China.

### 2.3. Animal Treatment

After subcutaneous inoculation of tumor cells, the activity, vigor, local reactions, and changes in tumor nodules were observed daily in the nude mice at approximately the same time of the day. At 2 to 3 days after inoculation, all the nude mice developed tumor nodules approximately 4-5 mm in size at the inoculation site. At 2 weeks after subcutaneous inoculation, the 12 nude mice survived and developed tumor nodules approximately 300 mm^3^ in size. The nude mice were then randomly assigned to the 100 mg/kg Xiaoaiping or 100 *μ*L/20 g normal saline (control) groups (n=3/sex/group). From the 14th day, we administered the mice to Xiaoaiping or normal saline for 18 consecutive days.

### 2.4. Tumor Growth in the Nude Mice following Treatment

The tumor size was measured on days 17, 20, 23, 26, 29, and 32. The tumor volume was calculated using the formula (A × B^∧^2)/2, where A is the longest diameter of the tumor and B is the shortest diameter of the tumor. The animals were euthanized on the 32nd day, and the tumor tissues were isolated for weighing.

### 2.5. qPCR Analysis of the mRNA Expression of Stemness-Related Genes in the Subcutaneous HCC Xenografts

Total RNA was extracted from the HCC tumor tissues using TRIzol in accordance with the manufacturer's instructions. The concentration and purity of RNA were determined using an ultraviolet spectrophotometer. Equal amounts of mRNA from each animal were reverse-transcribed into complementary DNA (cDNA). Quantitative PCR (qPCR) was performed to evaluate mRNA expression using the primers in Table 1. The qPCR conditions were predenaturation at 94°C for 30 seconds (s); 40 cycles of denaturation at 94°C for 30 s, annealing at 60°C for 30 s, elongation at 72°C for 60 s; and a final extension at 72°C for 10 min. We are sure that *β*-actin was used as the internal reference in the qPCR. Each reaction was performed three times in triplicate. The relative expression of the target genes was calculated using the 2^−ΔΔCt^ method.

### 2.6. Western Blot Analysis of the Protein Expression of Stemness-Related Genes in the Subcutaneous HCC Xenografts

The tumor tissues were lysed in radioimmunoprecipitation assay (RIPA) buffer containing protease inhibitors. The concentrations of the extracted proteins were determined using the bicinchoninic acid (BCA) method. Equal amounts of total protein from each animal were subjected to sodium dodecyl sulfate-polyacrylamide gel electrophoresis (SDS-PAGE) in 10-12% gels. After electrophoresis, the proteins were transferred onto the polyvinylidene fluoride (PVDF) membranes, which were incubated with the anti-CD90 antibody (Biorbyt Ltd., UK; 1:200 dilution), anti-notch1/2/3 antibodies (Beijing Biosynthesis Biotechnology Co., Ltd., China; 1:500 dilution), anti-CD13 antibody (Abcam, USA; 1:1000 dilution), anti-CD133 antibody (Abcam, USA; 1:2000 dilution), anti-EpCAM antibody (Proteintech Group, USA; 1:2000 dilution), and anti-ALDH1A1 antibody (Abcam, USA; 1:500 dilution) at 4°C overnight. Glyceraldehyde 3-phosphate dehydrogenase (GAPDH) was used as the internal reference in the western blot. The membranes were blocked with 5% skim milk for 2 hours and then incubated with the horseradish peroxidase-labeled anti-rabbit secondary antibody (1:3000 dilution; Biorbyt Ltd., UK). The proteins were visualized using a chemiluminescence method in a darkroom.

### 2.7. Statistical Analysis

Data were statistically analyzed using the SPSS 18.0 statistical analysis software, and the results are expressed as the mean ± standard deviation (x±s). Group comparison was conducted using the independent samples t-test. A P value less than 0.05 was considered statistically significant.

## 3. Results

### 3.1. Subcutaneous HCC Xenograft Growth in Nude Mice following Xiaoaiping Treatment

**Figure 1:**
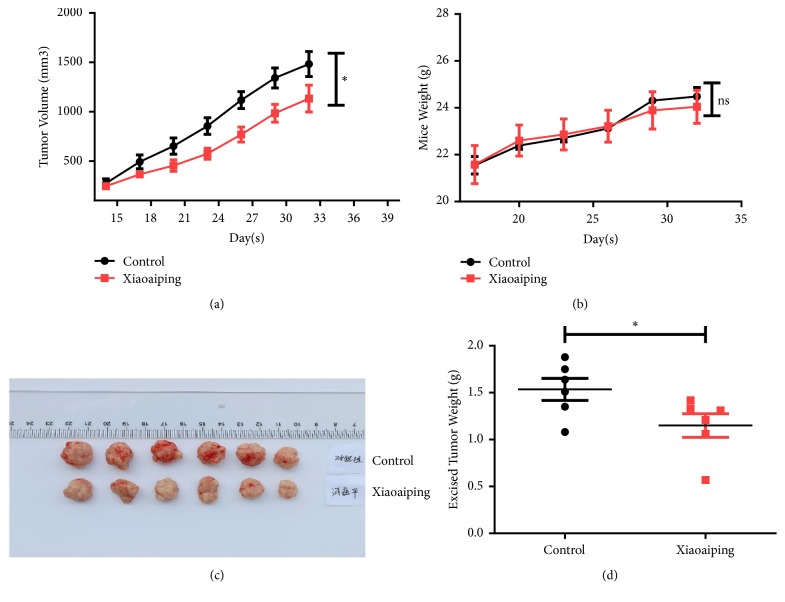
(a) HCC tumor volume changes over time under 100 mg/kg Xiaoaiping or normal saline (control) treatment. *∗* indicates P < 0.05 compared with the control group. (b) There was no significant difference in body weight between the two groups. (c) The images of the isolated tumors following 100 mg/kg Xiaoaiping or normal saline (control) treatment for 14 days. (d) The final tumor weights following 100 mg/kg Xiaoaiping or normal saline (control) treatment for 14 days. *∗* indicates P < 0.05 compared with the control group.

The tumor formation rate was 100% in the nude mice, with visible oval-shaped subcutaneous nodules at the inoculation sites. The nodules gradually became irregularly shaped with an uneven surface and lobular characteristics. As the tumor volume increased, the nude mice were sluggish and had decreased activity, appetite, and body weight. Compared to the control group, the subcutaneous xenograft tumors in the 100 mg/kg Xiaoaiping group significantly grew slower and were smaller in size (P<0.05; Figure 1(a)). There was no significant difference in body weight between the two groups (Figure 1(b)). The images of the final tumors are shown in Figure 1(c). The excised tumor weights were significantly lower in the Xiaoaiping group compared with controls (Figure 1(d)).

### 3.2. mRNA Expression of Stemness-Related Genes in the Subcutaneous HCC Xenografts in Nude Mice

**Figure 2:**
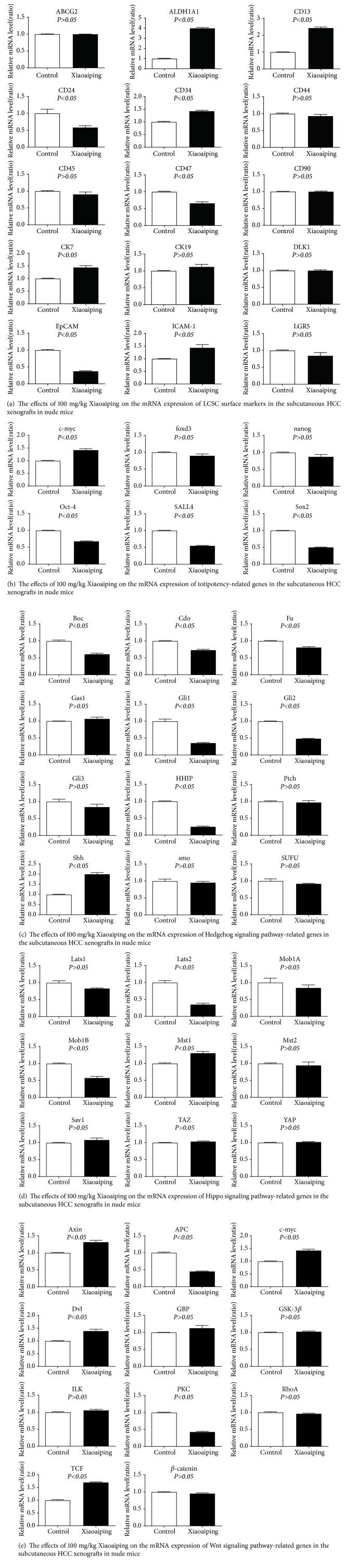


The Xiaoaiping effects on the mRNA expression of LCSC surface markers are shown in Figure 2(a). Xiaoaiping treatment resulted in the upregulation of CD13 and ALDH1A1, downregulation of CD24, EpCAM, and CD47, and no changes on the expression of CD133, CD90, cytokeratin 19 (CK19), delta like noncanonical Notch ligand 1 (DLK1), CD45, leucine-rich repeat-containing G-protein coupled receptor 5 (LGR5), ATP-binding cassette subfamily G member 2 (ABCG2), CD44, CD34, intercellular adhesion molecule 1 (ICAM-1), or cytokeratin 7 (CK7).

The Xiaoaiping effects on the mRNA expression of totipotency-related genes are shown in Figure 2(b). Xiaoaiping treatment resulted in the downregulation of Sox2, Oct-4, and SALL4 and no changes on the expression of c-myc, forkhead box protein D3 (foxd3), or nanog.

The Xiaoaiping effects on the mRNA expression of the genes related to the Hedgehog signaling pathway are shown in Figure 2(c). Xiaoaiping treatment led to decreased expression of hedgehog-interacting protein (HHIP), glioma-associated oncogene homolog 1 (Gli1), GLI family zinc finger 2 (GLI2), and brother of CDO (Boc), increased expression of sonic hedgehog (Shh), and no expressional changes in desert hedgehog (Dhh), hedgehog (Hh), Ptch, smoothened (Smo), GLI family zinc finger 3 (GLI3), growth arrest-specific 1 (Gas1), suppressor of fused (Sufu), fused (Fu), or cysteine dioxygenase (Cdo).

The Xiaoaiping effects on the mRNA expression of the genes related to the Hippo signaling pathway are shown in Figure 2(d). Xiaoaiping treatment caused decreased MOB kinase activator 1B (Mob1B) expression and no expressional changes in mammalian STE20-like kinase 1 (Mst1), mammalian STE20-like kinase 2 (Mst2), large tumor suppressor kinase 1 (Lats1), large tumor suppressor kinase 2 (Lats2), salvador family WW domain containing protein 1 (Sav1), MOB kinase activator 1A (Mob1A), Yes-associated protein 1 (YAP), or tafazzin (TAZ).

The Xiaoaiping effects on the mRNA expression of the genes related to the Wnt signaling pathway are shown in Figure 2(e). Xiaoaiping treatment resulted in the downregulation of adenomatous polyposis coli (APC) and Wnt, upregulation of T-cell factor (TCF) and lymphoid enhancer-binding factor (LEF), and no expressional changes in axin, DVL, frizzled (FZ), guanylate-binding proteins (GBP), c-myc, *β*-catenin, glycogen synthase kinase 3 beta (GSK-3*β*), integrin linked kinase (ILK), or casein kinase 1 (CK1).

### 3.3. Protein Expression of Stemness-Related Genes in the Subcutaneous HCC Xenograft Tissues in Nude Mice

**Figure 3:**
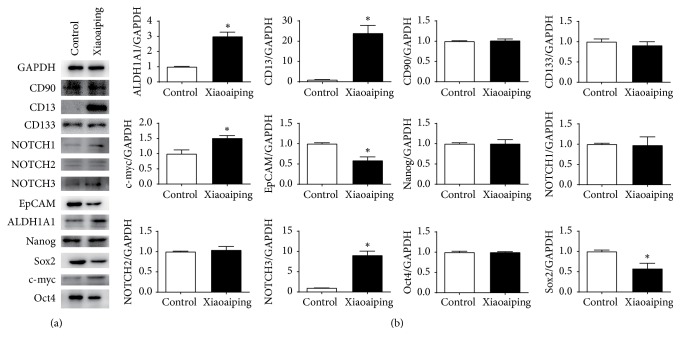
The effects of 100 mg/kg Xiaoaiping on the protein expression of HCC stemness-related genes. *∗* indicates P < 0.05 compared with the control group.

Western blot analysis was performed to examine the effects of 100 mg/kg Xiaoaiping on the protein expression of the stemness-related genes. Xiaoaiping upregulated the expression of CD13 and ALDH1A1 proteins but downregulated the expression of EpCAM, Sox2, and Oct4 proteins (Figures 3(a) and 3(b)). The western blot results were consistent with the qPCR results.

## 4. Discussion

Liver cancer is a highly malignant solid tumor with high incidence and poor prognosis. Marsdenia tenacissima has been used for thousands of years in China and has now been reported for its anticancer effects in a variety of tumors. Xiaoaiping is the extract from Marsdenia tenacissima and has been approved in China for the treatment of advanced liver cancer alone or in combination with radiotherapy and chemotherapy [11]. The anticancer component of Marsdenia tenacissima is a C21 steroidal saponin [13]. Recently, the molecular mechanisms of the anticancer effects of Marsdenia tenacissima have been revealed. Marsdenia tenacissima reduces the multidrug resistance mainly through modulating K-ras, P-pg, c-Met, EGFR, Axl, MRP-1, BCRP, and other relevant genes. Marsdenia tenacissima inhibits tumor growth via the modulation of VEGF, MMP-2, MMP-9, GSH-Px, SOD, CAT, and MDA. Marsdenia tenacissima induced cell cycle arrest (G0/G1) via the influence on Cyclin D1. Marsdenia tenacissima modulates the CCR5-CCL5 axis, FAK, and RhoC genes that are involved in tumor invasion and metastasis. Marsdenia tenacissima induced apoptosis in tumor cells by regulating the Caspase 3, Caspase 9, Bak, Bax, Bcl-xl, Bcl-2, Fas, and PKC genes, as well as the PI3K/AKT/mTOR and the Hippo signaling pathways. Furthermore, Marsdenia tenacissima inhibits tumor cell proliferation via regulating the VEGF/VEGFR, MARK, and ERK1/2 genes and the PI3K-PTEN-mTOR and Hippo signaling pathways [14].

In recent years, several studies have demonstrated that LCSCs are important for liver cancer cell proliferation, differentiation, recurrence, and drug resistance. Recent studies have indicated that the antitumor effect of Marsdenia tenacissima is related to the stemness of the liver cancer cells. Zhang et al. showed that Xiaoaiping inhibits the proliferation and induces the apoptosis of HCC cells, which may be achieved by inhibiting the Hippo signaling pathway [15].

Our study found that Xiaoaiping downregulated the mRNA expression of the Hedgehog signaling pathway-related genes, including HHIP, Gli1, Gli2, and Boc, but upregulated the expression of Shh. With regard to the mRNA expression of genes involved in the Hippo and Wnt signaling pathways, Xiaoaiping downregulated the expression of Mob1B, APC, and Wnt but upregulated the expression of TCF and LEF. Examination of the protein expression yielded similar results. Collectively, these results suggested that Xiaoaiping suppresses the growth of HCC by inhibiting multiple stemness pathways, including the Hedgehog and Hippo signaling pathways.

The Hippo signaling pathway was first discovered in* Drosophila melanogaster*. In humans, the key molecules in the Hippo signaling pathway include YAP, Mst, and Lats. The overexpression of YAP and TAZ is essential for tumor proliferation, differentiation, and metastasis. In contrast, Mst1 and Mst2 play a negative regulatory role in tumor proliferation. The low expression of the tumor suppressor gene, Lats, is a marker of active malignancy [16]. Our data showed that Xiaoaiping increased Mst1 expression but had no effect on Lats1 expression, indicating that Xiaoaiping exerts the antitumor effect through activating Mst1.

The Hedgehog signaling pathway is involved in HCC cell autophagy, proliferation, and apoptosis. A prior study has shown that inhibition of Gli1 and Gli2 significantly enhances autophagy, promotes apoptosis, and reduces viability of HCC cells [17]. Our study found that Xiaoaiping significantly reduced the expression of multiple Hedgehog pathway-related genes, including Gli1 and Gli2. Therefore, the inhibitory effect of Xiaoaiping on tumor growth may be achieved by enhancing the autophagy and apoptosis of HCC cells, as well as reducing its cellular activity via the downregulation of Gli1 and Gli2. A previous study has shown that hepatitis B virus X protein (HBx) increases the HCC occurrence by stimulating the expression of Hedgehog signaling pathway-related genes in human HCC cells [18]. The cell proliferation and migration stimulated by HBx are mediated by the activation of the Hedgehog pathway (especially the Gli1 gene) [19]. The inhibition of the Hedgehog pathway by Xiaoaiping may result in anti-HBx effects, which warrants further investigation given that the Chinese liver cancer patients are mostly HBx-positive.

It is worth noting that the stem markers CD13 and ALDH1A1 were upregulated by Xiaoaiping treatment. CD13 is one of the key stem markers and is related to the proliferation, metastasis, and angiogenesis of liver cancer cells. CD13+ LCSCs are in the G0/G1 phase and are the most abundant side population in the liver cancer. CD13 is highly expressed in the quiescent phase of cells and plays a role in ATP transporter-related multidrug resistance [20]. In this study, CD13 expression was increased by Xiaoaiping, indicating that there may be a cell population resistant to Xiaoaiping and that survived after the treatment. Yang et al. have found that high expression of ALDH1A1 is associated with partial liver cancer recurrence [21]. Therefore, although Xiaoaiping inhibited HCC growth, it may lead to drug resistance in some HCC cells.

In conclusion, our study demonstrated that Xiaoaiping was effective to treat advanced liver cancer and significantly reduced the growth of subcutaneous HCC xenografts in nude mice. The anticancer effect of Xiaoaiping may be mediated by modulating the expression of multiple stemness markers and totipotency factors and inhibition of the Hippo, Wnt, and Hedgehog signaling pathways. This study provided the molecular mechanism to support the use Xiaoaiping in the treatment of advanced liver cancer.

## Figures and Tables

**Table 1:** Sequence of the primers used in qPCR.

Primer name	Forward sequence5′-3′	Reverse sequence3′-5′	Products (bp)
PTCH	TGGAACGAGGACA AAGCGG	AGGCATAGGCGAGCATGAGTAA	202
SMO	TCTCGGGCAAGACCTCCTACT	CGCACGGTATCGGTAGTTCTT	146
GLI1	CCAAGCACCAGAATCGGACC	TTTGGTCACATGGGCGTCAG	140
GLI2	AGGGATGACTGTAAGCAGGAGG	CGGCACACAAACTCCTTCTTCT	152
GLI3	GCACTAAGCGTTACACAGACCCA	CTTCTCTGCCTTGACGGTTTTC	257
SHH	CTGCTGGTATGCTCGGGACT	CATTGGGGATAAACTGCTTGTAG	106
DHH	GATGACCGAGCGTTGTAAGGA	GTTATCAGCTTTGACCGACACG	277
FU	TTGCTGCCCAGTTGGTGTCA	TGTGGTCGTATGGTCGCTCCT	225
GAS1	ATCTGCGAGTCGGTCAAGGA	TGCGCTGCTCGTCATCGTA	133
Cdon	CTTTTCCAGCCGTCCAATAACT	GTGCCACTGCTTTGAACCTTCT	205
BOC	CATCACTGCCCTTAACAACCAC	TGTTTCCCTCATCCACTTCAATC	158
HH	TCACCGTCTGGCACCCTAGT	CATGGCTCCTCTTGAACCCTG	120
HHIP	GAGAAGGTGCCTGAATGGGAAC	GGTGAGTGGAACAGGCTTTGA	270
SUFU	GCCTTCGCTTCGCTCTTTC	AGGCCGAAGCTGATGTAGTGC	212
MST1	CGGGTCCCAGTAGCCAAGAT	GTGTCATTACCCGTACCTTTGG	191
MST2	ACCATCTGCCTTAGGAACGGA	TAATTGCGACAACTTGACCGG	223
LATS1	CCTATTAATGCCAGCATGAAACC	CGTTGCTAGGGTGAGCTTGA	220
LATS2	GAGCAGATTGTGCGGGTCATT	TGGTGGTAGGACGCAAACGA	116
SAV1	GAATGCCACAGAATCAGGGGA	GATGCCTGTATTGGGCCTTCTT	291
MOB1A	AGTGGGAATCTGAGACAAGCTG	GGTGCAGAACATTTGATTGGCT	224
MOB1B	TGGGCAGATGGAACGAACA	GAAGCTGGATCACAGGGTCAA	232
YAP	GAGTTAGCCCTGCGTAGCCA	GGCAGGGTGCTTTGGTTGATA	272
TAZ	CCTGAAACTCCGCCACATCT	CTGGTAGACGCCATCTCCTTTC	235
Notch1	GTCAACGCCGTAGATGACC	TTGTTAGCCCCGTTCTTCAG	101
Notch2	ACTGTGAGGAGCAACTCGAT	TCCACTTCATACTCACAGTTGA	133
Notch3	TGACCGTACTGGCGAGACT	CCGCTTGGCTGCATCAGCA	67
Notch4	AACTCCTCCCCAGGAATCTG	CCTCCATCCAGCAGAGGTT	168
JAG1	GCCAGGAAGTTTCAGGGAGA	GCTGGAGACTGGAAGACCGA	264
JAG2	CTGACTGCCGCATCAACATC	GCTACAGCGATACCCGTTGA	92
DLL1	TCTCCTGATGACCTCGCAACA	TCACACACGAAGCGGTAGGAG	149
DLL3	CACTCAACAACCTAAGGACGCA	CGAGGAAGGGTAGGGAAAAAG	212
DLL4	GACCTCTCCACAGACACCTTTG	TCCACTTCCAGCTCCTTCTTCT	299
HES1	ATTCTGGAAATGACAGTGAAGCAC	CACCTCGGTATTAACGCCCTC	168
HES5	GAAAAACCGACTGCGGAAGC	GACGAAGGCTTTGCTGTGCT	184
NGN	CAGATTTGGTCCCATTTGTGAG	ATGGCAACACTACATCCTGACC	279
Hey1	GAAGCAGGTAATGGAGCAAGGA	GAAGCGTAGTTGTTGAGATGCG	258
HEY2	AAGGCTACTTTGACGCACACG	GAGATGAGACACAAGCCGCAC	146
HES1	ATTCTGGAAATGACAGTGAAGCAC	CACCTCGGTATTAACGCCCTC	168
DV1	AGAAGTCAGCTCTTGCCTCAGTT	ATCTCATCAGTAGCACGACGAAG	256
AXIN	TCTGGATACCTGCCGACCTTA	TCTGCTGCTCGCTGTCGTT	271
APC	CAAAACTGGAAACTGAGGCATCT	ACTCTCCAGAACGGCTTGATACA	225
*β*-catenin	GCCAAGTGGGTGGTATAGAGG	GGGATGGTGGGTGTAAGAGC	192
GSK3B	GTTAGCAGAGACAAGGACGGCA	GCAATACTTTCTTGATGGCGAC	183
GBP	AGGTGGCTCCTGACGCTAA	CAGGCTGGAAGGGAAAGACA	180
TCF	GAGTATGCCTACCTCAAAGCCA	AGCCGCTTGATCTTCCCTG	80
FRZB	ACGGAAACTGTAGAGGGGCA	CAGTGTCCCGTGGAATGTTTAC	204
c-Myc	TGCTGCCAAGAGGGTCAAGT	GCTCCGTTTTAGCTCGTTCC	160
PKC	GCTTATGCTGTCATGTCCCGG	GATGATGAGGACTCCCCCCA	120
RHOA	ATTGTTGGTGATGGAGCCTGTG	GTGGGCACATACACCTCTGG	83
ILK	GCAGTGAATGAACACGGGAAT	CACCAGGTCCTCTGCCACTT	78
CD24	GCTCCTACCCACGCAGATTT	GGTGGCATTAGTTGGATTTGG	109
CD13	CCGACATTGACAAGACTGAGCT	ACCTTTCTGACATTGCCCTCC	175
CD44	TCCAACACCTCCCAGTATGACA	CTTTCTGGACATAGCGGGTG	151
CD90	CGCTCTCCTGCTAACAGTCTTG	GTTCGGGAGCGGTATGTGTG	213
CD45	TGAGGAGCAAGGAAGCCAATC	GCCACCAACTGAAGGCTGAAC	77
ALDH1	TGCCGACTTGGACAATGCT	GCCTCCTCCACATTCCAGTTT	277
ICAM1	CCGTTGCCTAAAAAGGAGTTGC	TGGCAGCGTAGGGTAAGGTTC	221
LGR5	CGGGAAACGCTCTGACATACAT	ACTTCTAAAAGCCTGGACGGG	260
CK7	TGAAGAAGGATGTGGATGCTGC	CAGCTCTGTCAACTCCGTCTCAT	119
CK19	AGAATTGAACCGGGAGGTCG	CCTGATTCTGCCGCTCACTA	257
CD34	GCAACATCTCCCACTAAACCCTA	GTCCTTCTTAAACTCCGCACAGC	156
CD47	ACCTCCTTCGTCATTGCCATATT	ATACACGCCGCAATACAGAGAC	89
DLK1	CCCTTTGTGACCAGTGCGTG	ATTCATAGAGGCCATCGTCCAG	183
EpCAM	TAATCGTCAATGCCAGTGTACTTC	AGCCATTCATTTCTGCCTTCAT	101
CD133	TGGCATCTTCTATGGTTTTGTGG	TCCTTGGTAGTGTTGTACTGGGC	159
OCT4	GAGTGAGAGGCAACCTGGAGAAT	ACCGAGGAGTACAGTGCAGTGAA	292
SOX2	GGTTACCTCTTCCTCCCACTCC	CGCTCTGGTAGTGCTGGGAC	140
NANOG	GAGAAGAGTGTCGCAAAAAAGGA	TGAGGTTCAGGATGTTGGAGAGT	163
FOXD3	GCAACTACTGGACCCTGGAC	TAAGCGCCGAAGCTCTGCAT	142
SALL4	CATCAACTCGGAGGAGGACCA	TGATGAGGACAGGTGGATTTTTAGT	275
*β*-actin	AGTTGCGTTACACCCTTTCTTGAC	GCTCGCTCCAACCGACTGC	171

## Data Availability

All data generated or analyzed during this study are included in this published article.
